# Physical activity and medicine use: evidence from a population-based study

**DOI:** 10.1186/1471-2458-6-224

**Published:** 2006-09-06

**Authors:** Andrea D Bertoldi, Pedro C Hallal, Aluisio JD Barros

**Affiliations:** 1 Post-Graduate Program in Epidemiology – Federal University of Pelotas, Brazil Av. Duque de Caxias, 250 - 3^rd ^floor, 96030-002, Pelotas, RS, Brazil

## Abstract

**Background:**

Few studies have investigated the association between physical activity practice and medicine use; data from these studies are inconsistent. The aim of this study was to evaluate the association between level of physical activity and medicine use in adults aged 20 years or more.

**Methods:**

A population-based cross-sectional study was carried out in the first semester of 2002 in the urban area of Pelotas; a medium-sized Southern Brazilian city. Physical activity was assessed with the short version of the International Physical Activity Questionnaire. A physical activity score was created as the weekly time spent in moderate-intensity activities plus twice the weekly time spent in vigorous-intensity activities. Medicine use in the 15 days prior to the interview was also assessed. Adjusted analyses taking into account the sampling design was carried out using Poisson regression. Wald tests for heterogeneity and linear trend were used to calculate significance.

**Results:**

Out of the 3,182 individuals interviewed, 41% were not sufficiently active according to current physical activity guidelines. Only 34% of the subjects did not use medicines in the previous 15 days, and 18% used three or more drugs in the same period. Level of physical activity was inversely associated with the number of medicines used both in the crude and in the adjusted analyses.

**Conclusion:**

There are well-documented benefits of physical activity for several chronic diseases in the literature. Data from the present study suggest that medicine use is also positively affected by physical activity behavior.

## Background

Lack of physical activity is now recognized as a major challenge for public health due to its association with several chronic diseases and consequent premature mortality [1-6]. The World Health Organization has included physical activity in the health agenda by creating the Global Strategy on Diet, Physical Activity and Health [7]. However, assessment of physical activity has also been a challenge. In 2003, a group of researchers and institutions proposed the International Physical Activity Questionnaire (IPAQ)[8], allowing investigators to carry out comparable studies on physical activity.

Medicine use is an indicator of general health status [9], and consequently, less active individuals may be more likely to use medicines. However, the literature on this association is scarce. Bardel et al[10], studying women aged 35–65 years, showed a higher frequency of medicine use in the less active group in the crude analysis. However, after adjusting for the confounding variables, the association was no longer significant. Eggen et al[11] found no association between leisure-time physical activity and medicine use in Norway, in a study including adolescents and adults.

In a population-based study of Brazilian adults, the association between level of physical activity and medicine use was evaluated, carefully controlling for potential confounders, such as sex, age and economic status, and mediators, such as body mass index (BMI) and self-reported health status.

## Methods

A cross-sectional population-based study was carried out in the first semester of 2002 in the urban area of Pelotas, a 320,000-inhabitant city in Southern Brazil. The sample was selected in two stages. The primary sample units were the census tracts; these were divided into four groups according to the mean schooling level of the household heads. Within each stratum, the census tracts were systematically selected with probability proportional to size. A total of 1,600 households were selected, 20 in each of the 80 tracts, through systematic sampling. All residents of the selected household aged 20 years or more were eligible for the investigation.

The sample size obtained (n = 3,182) allowed us to estimate the association between medicine use and physical activity level with a power of 90% using the following parameters: confidence level of 95%, prevalence of medicine use in the 15 days prior to the interview of 66%, prevalence of sedentary lifestyle of 27%, and prevalence ratio of 1.15 or above.

A pre-tested and standardized questionnaire was used to assess medicine use, with a recall period of 15 days. Interviewees were asked to show the prescriptions and packaging of all medicines used in the period. The name of the drug and the manufacturer were recorded. Physical activity was measured using the short version of the International Physical Activity Questionnaire (IPAQ), with a weekly recall[8]. A physical activity score was created as the weekly time spent in moderate-intensity activities plus twice the weekly time spent in vigorous-intensity activities. The score was categorized into four groups – 0 minutes per week: inactive; 1–149 minutes per week: insufficiently active; 150–999 minutes per week: sufficiently active; ≥ 1000 minutes per week: highly active. The two least active groups do not reach current physical activity requirements of at least 150 minutes per week of moderate-intensity physical activities[3,5].

Demographic and socioeconomic variables were also collected as potential confounders. These variables were age, sex, and economic status (according to the Brazil Criterion for Economic Classification proposed by ANEP[12], which considers both household assets and education of the household head). Families are classified from class A (wealthiest) to E (poorest). Body mass index – BMI (kg/m^2^) and self-reported health status (excellent, very good, good, average, or poor) were also assessed.

Interviewers were women with at least 11 years of formal education. They were trained for 40 hours and were not aware of the purposes and hypotheses of the investigation. Data were collected through face-to-face home interviews. For quality control purposes, 10% of the interviews were repeated by field supervisors.

Following descriptive analyses, the crude association between medicine use and physical activity was tested. In order to adequately adjust for confounders, Poisson regression was used[13]. The effect of physical activity on medicine use was initially adjusted for the potential confounding effect of sex, age, and economic status. Thereafter, additional adjustment for mediators (BMI and self-reported health status) was also carried out. All tests were two-tailed, and the significance level used was 0.05. All analyses were performed accounting for the sample design by using the *svy *set of commands available in Stata version 8 (Statacorp, College Station, TX, 2003). The census tracts were defined as the primary sampling units, and weighting was not necessary, given the fact that the sample was self-weighted.

The project was approved by the Ethics Committee of the Federal University of Pelotas Medical School. Personal data were made anonymous, and informed consent was obtained from each subject prior to the interview.

## Results

In the 1,600 selected households, there were 3,372 individuals eligible for the study, of whom 3,182 (94.4%) answered the questionnaire. Analyses were carried out using 3,119 observations, as 63 subjects provided incomplete data on physical activity. The design effects for medicine use and physical activity were, respectively, 2.9 and 4.7, with intra-class coefficients of 0.046 and 0.094, respectively.

Table 1 describes the sample in terms of sex, age, economic status, BMI, self-reported health status, medicine use and physical activity. There were slightly more women than men in the sample. The mean age was 44.0 years (SD 16.3; range 20–98). Most subjects were classified in the intermediate economic group. The mean BMI was 25.5 kg/m^2 ^(SD 4.6) and only 9.5% of the individuals reported their health status to be excellent. Approximately 25% of all subjects were classified as inactive in the week prior to the interview. Further 443 (14.2%) practiced some physical activity in the week prior to the interview, but did not reach current guidelines. Only 34.1% of the subjects did not use medicines in the 15 days prior to the interview, while 579 (18.2%) used three or more medicines in the same period.

**Figure 1:**
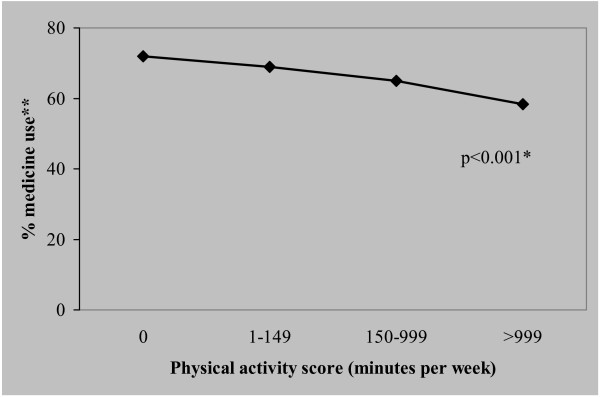
Prevalence of medicine use in the 15 days prior to the interview according to physical activity categories. Pelotas, Brazil, 2002. * Wald test for trend. ** Percentage of any medicine use in the 15 days prior to the interview.

Figure 1 shows that the higher the level of physical activity, the lower the frequency of medicine utilization (P < 0.001). The proportions of medicine use were 71.9%, 68.9%, 65.0% and 58.4% among inactive, insufficiently active, sufficiently active and very active individuals, respectively.

**Figure 2:**
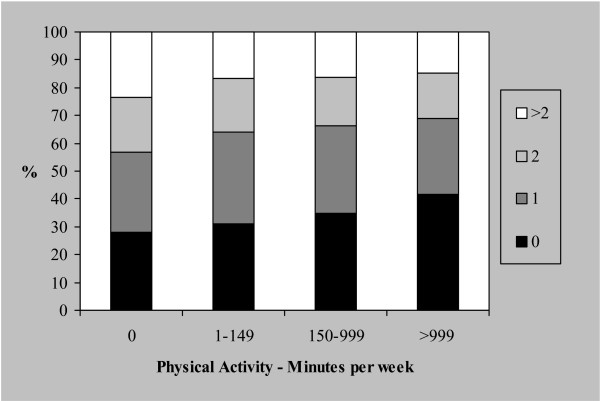
Association between physical activity practice and number of medicines used in the 15 days prior to the interview. Pelotas, Brazil, 2002.

Figure 2 presents the relationship between the number of medicines used and level of physical activity. The proportion of individuals using three or more drugs was higher among inactive subjects (P < 0.001). High level of physical activity was associated with lower medicine utilization.

Table 2 shows the association between physical activity categories and medicine use in the 15 days prior to the interview, categorized as a yes/no variable. In the crude analysis, there was an inverse association between activity levels and medicine use. After adjustment for sex, age and economic status, the effect was reduced, but remained statistically significant. Inactive and insufficiently active subjects presented similar adjusted prevalence ratios. Further adjustment for the mediating effects of BMI and self-reported health status did not substantially modify the results.

For comparison with a previously published study [10], we have repeated analysis exclusively for women aged 35–65 years. For this group, the increased risk for medicine use among sedentary women was 23%.

Data on medical diagnosis of diabetes and hypertension was collected among subjects aged 40 years or more. Based on this information, we repeated the analysis for individuals reporting either condition. Among diabetic patients (N = 219), 86.1% of the sedentary individuals were using medicines, compared to 64.5% among those in the more active group (P = 0.045). The same pattern was observed for hypertensive patients (N = 737). The prevalence of medicine utilization was 87.7% among sedentary patients, compared to 79.9% among the more active subjects (P = 0.09). These results did not change after adjustment for age.

## Discussion

To the best of our knowledge, this study is the first to report that physical activity practice is associated with a decreased risk of medicine use even after adjusting for confounders. Because both physical inactivity and medicine use are highly prevalent, the increased risk of medicine use among subjects who do not reach physical activity guidelines is relevant for public health, even with the low prevalence ratios detected. Rates of physical inactivity and medicine use in our sample were consistent with previous Brazilian reports [14-16].

A Swedish study[10] including only middle-aged (35–65 years) women evaluated the association between occupational and leisure-time physical activity and medicine use. In the crude analysis, sedentary individuals showed 39% higher medicine use in comparison to very active women. In our study, the equivalent percentage was 23%. However, after adjusting for self-reported health status, BMI, age and educational level, the association between activity levels and medicine use failed to reach significance in the Swedish study[10]. In our analysis (Table 2), the effect of physical activity on medicine use remained significant after adjustment for sex, age and economic status (model 1), and after inclusion of BMI and self-reported health status (model 2). A study in Norway[11] found no relationship between leisure-time physical activity and medicine use. The fact that our study addressed all components of physical activity (occupational, commuting, leisure-time and household chores), and those mentioned above evaluated only fractions of it, might explain the different findings. If physical activity really affects medicine use, studies focusing on the whole behavior, such as ours, may be more likely to detect the association than those evaluating only fractions of it.

A recall period of 15 days was used to assess medicine utilization, and thus, some degree of recall bias is possible[17]. Even so, 15 days (or two weeks) is the most frequent recall period used in the literature[11,18-21]. Among the methodological strengths of the study, the low non-response rate (5.6%) and the similarity between the sample and the whole city census data (data not shown) should be noted. The measurement of the total amount (occupational, commuting, leisure-time and household chores) of physical activity, instead of fractions of it, should also be highlighted.

In previous publications [18,22], we have shown that both medicine use and lack of physical activity were strongly associated with poor self-reported health status in this sample. However, these two variables are differently correlated with health. While physical activity has an important role in the prevention of unhealthy outcomes [1-6], medicine use is often a consequence of health status.

The initial hypothesis of our study was that physical activity should affect medicine utilization patterns through health status. However, our association was still significant after adjusting for self-reported health status. Therefore, the effect of physical activity practice on medicine utilization is partially independent of self-reported health status. We do not have a definitive explanation for this finding, but some hypotheses may be presented. First, although the association between physical activity and medicine use was significant, one should bear in mind that the cross-sectional design used may lead to reverse causality bias. Therefore, the hypothesis presented in this paper should be confirmed in prospective studies. Second, medicine use is not determined only by pharmacological factors. There are well-known variables which influence medicine use, related to social, anthropological, behavioral and cultural aspects [23]. Thus, more active individuals may have different behavioral and cultural patterns, and be less likely to be influenced by these external factors which increase the likelihood of medicine use. Third, the missing impact of self-reported health status on the association between physical activity and medicine use, might be due to the pharmacological effect of medicine use on the perception of symptoms and health. Future studies may help understand this finding.

One should argue that the significant association described here is consequence of residual confounding. However, the fact that the adjusted analysis did not provide results substantially different from those obtained in the crude one, minimizes the likelihood of residual confounding. Another methodological aspect to be considered is that our conclusions are not based only on crude results; actually we have carried out two regression models and the association was confirmed in both. In the first model, we intended to remove the confounding effect of demographic and socioeconomic indicators. Thereafter, we have also adjusted for the mediating effect of BMI and self-reported health status, which are strongly associated both with the exposure and the outcome.

## Conclusion

In summary, level of physical activity was inversely associated with the prevalence and number of medicines used in a population-based sample of adult Brazilians, although prospective studies are warranted in order to confirm temporality for this association. Therefore, it is possible that strategies aimed at increasing population levels of physical activity may help decrease the high rates of medicine use observed both in low-, middle- and high-income countries[10,11,18,19,24]. The fact that this association was confirmed among subjects with chronic diseases highlights its importance. It means that even after the disease is present, active individuals are less likely to use medicines.

## Competing interests

The author(s) declare that they have no competing interests.

## Authors' contributions

ADB coordinated the fieldwork and was responsible for the collection of medicine utilization data. PCH also coordinated the fieldwork and was responsible for the collection of physical activity data. Data analyses were coordinated by ADB and PCH. AJB supervised all phases of the study. All authors were responsible for writing, and approved the final version of the manuscript.

## Pre-publication history

The pre-publication history for this paper can be accessed here:



## Figures and Tables

**Table 1:** Description of the sample in terms of demographic and socioeconomic variables, physical activity and medicine use. Pelotas, Brazil, 2002.

Variable	N	Percentage
Sex		
Male	1374	43.2%
Female	1808	56.8%
Age (years)		
20–39	1399	44.0%
40–59	1200	37.7%
≥ 60	583	18.3%
Economic status *		
A – B (wealthiest)	747	23.6%
C	1270	40.0%
D – E	1153	36.4%
Body mass index (kg/m2)		
<25	1542	50.6%
25–30	1068	35.1%
≥ 30	437	14.3%
Self-reported health status		
Excellent	300	9.5%
Very good	480	15.2%
Good	1494	47.2%
Average	767	24.2%
Poor	125	4.0%
Physical activity score (min/week)		
0	839	26.9%
1–149	443	14.2%
150–999	1136	36.4%
≥ 1000	701	22.5%
Number of medicines used in the previous 15 days		
0	1084	34.1%
1	943	29.7%
2	571	18.0%
≥ 3	579	18.2%

* Brazil Criterion for Economic Classification (ANEP, 1996)

**Table 2:** Crude and adjusted prevalence ratios (PR) of medicine use in the 15 days prior to the interview according to physical activity score. Pelotas, Brazil, 2002.

VARIABLE	CRUDE ANALYSIS		ADJUSTED ANALYSIS*		ADJUSTED ANALYSIS**	
	
	PR (CI95%)	P value	PR (CI95%)	P value	PR (CI95%)	P value
Physical activity score		<0.001***		0.003***		0.005***
0 minutes/week	1.23 (1.12; 1.36)		1.13 (1.04; 1.24)		1.13 (1.03; 1.23)	
1–149 minutes/week	1.18 (1.06; 1.31)		1.14 (1.04; 1.26)		1.14 (1.04; 1.26)	
150–999 minutes/week	1.11 (1.01; 1.22)		1.07 (0.98; 1.17)		1.08 (0.99; 1.18)	
≥ 1000 minutes/week	1.00		1.00		1.00	

* Adjusted for sex, age and socioeconomic level

** Adjusted for sex, age, socioeconomic level, body mass index and self-reported health status

*** Wald test for trend
